# Detecting Validated Intracellular ROS Generation with ^18^F-dihydroethidine-Based PET

**DOI:** 10.1007/s11307-021-01683-0

**Published:** 2021-11-24

**Authors:** Edward C. T. Waters, Friedrich Baark, Zilin Yu, Filipa Mota, Thomas R. Eykyn, Ran Yan, Richard Southworth

**Affiliations:** 1 Division of Imaging Sciences and Biomedical Engineering, Kings College London, The Rayne Institute, St Thomas Hospital, London, SE1 7EH UK; 2 Center for Infection and Inflammation Imaging Research, Center for Tuberculosis Research, and Department of Pediatrics, Johns Hopkins University School of Medicine, Baltimore, MD 21287 USA

**Keywords:** Reactive oxygen species, ROS, Oxidative stress, Molecular imaging, PET

## Abstract

**Purpose:**

To determine the sensitivity of the ^18^F-radiolabelled dihydroethidine analogue ([^18^F]DHE) to ROS in a validated *ex vivo* model of tissue oxidative stress.

**Procedures:**

The sensitivity of [^18^F]DHE to various ROS-generating systems was first established *in vitro*. Then, isolated rat hearts were perfused under constant flow, with contractile function monitored by intraventricular balloon. Cardiac uptake of infused [^18^F]DHE (50–150 kBq.min^−1^) was monitored by γ-detection, while ROS generation was invoked by menadione infusion (0, 10, or 50 μm), validated by parallel measures of cardiac oxidative stress.

**Results:**

[^18^F]DHE was most sensitive to oxidation by superoxide and hydroxyl radicals. Normalised [^18^F]DHE uptake was significantly greater in menadione-treated hearts (1.44 ± 0.27) versus control (0.81 ± 0.07) (*p* < 0.05, *n* = 4/group), associated with concomitant cardiac contractile dysfunction, glutathione depletion, and PKG1α dimerisation.

**Conclusion:**

[^18^F]DHE reports on ROS in a validated model of oxidative stress where perfusion (and tracer delivery) is unlikely to impact its pharmacokinetics.

**Supplementary Information:**

The online version contains supplementary material available at 10.1007/s11307-021-01683-0.

## Introduction

The non-invasive detection of reactive oxygen species (ROS) by positron emission tomography (PET) imaging is an attractive prospect for many disease states including cancer, cardiovascular disease, and inflammatory and neurological conditions, where it might be exploited for early diagnosis, patient staging, and stratification, gauging response to therapy, or identifying drug toxicity [1–4].

While many imaging strategies have been explored for these applications, none is yet suitable for clinical use. A widely evaluated approach involves the repurposing of the well-established ROS-sensing fluorophore dihydroethidine (DHE), or its analogues. DHE is an uncharged blue fluorescent molecule which forms a red fluorescent cationic species when oxidised by ROS [5]. While the reduced molecule is sufficiently lipophilic to non-selectively penetrate cell membranes, the oxidised cation is not, suggesting that it may become selectively trapped inside cells overproducing ROS. It has been proposed that radiolabelled analogues of these fluorophores could therefore be used to non-invasively image oxidative stress by PET instead, harnessing its advantages in sensitivity, signal quantification, and tissue depth independence [4, 6, 7].

While early *in vitro* evaluation of radiolabelled DHE analogues appears promising, validation of these tracers *in vivo* is challenging. The animal models utilised to generate ROS for this purpose are often extreme and likely invoke vasoconstriction, vasodilation, microvessel occlusion, inflammation, oedema, and changes in vascular or cellular permeability, all of which potentially impact upon tracer pharmacokinetics and make it difficult to confirm the specificity of these probes to intracellular ROS overproduction.

We have constructed an apparatus which allows us to track the fate of injected radiotracers in isolated perfused rodent hearts in real time. It can be used to validate radiotracer behaviour with high levels of experimental control (in terms of perfusion, oxygenation, workload, energy substrate availability, or drug co-administration), monitoring cardiac haemodynamics, contractile performance, and metabolism while retaining the opportunity for post hoc validation using histology, enzyme assays, autoradiography, or Western blotting, *etc*. as required [8–13].

**Fig. 1:**
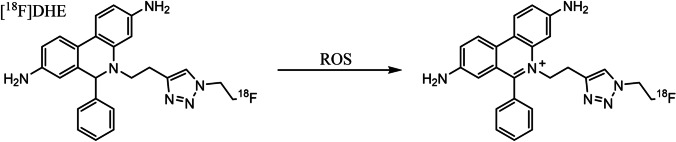
Structure of [^18^F]DHE and cation formation upon oxidation by ROS.

In this study, we have harnessed the advantages of this approach to induce controllable intracellular ROS in intact beating rat hearts perfused at a constant coronary flow rate (thereby minimising the confounding effects of regional variations in radiotracer delivery and washout) to establish the sensitivity and selectivity of the [^18^F]-radiolabelled DHE analogue 5-(2-(1-(2-[^18^F]fluoroethyl)-1H-1,2,3-triazol-4-yl)ethyl)-6-phenyl-5,6-dihydrophenanthridine-3,8-diamine ([^18^F]DHE) (Fig. 1), to tissue ROS overproduction.

## Methods

### Reagents and Gas Mixtures

All reagents were purchased from Sigma-Aldrich unless otherwise stated. All gas mixtures were purchased from BOC Industrial Gases.

### Animals

Male Wistar rats (280–320 g, Charles River, UK) were used for all experiments. All experimental procedures were approved by King’s College London’s local Animal Care and Ethics Committee and carried out in accordance with Home Office regulations as detailed in the Guidance on the Operation of Animals (Scientific Procedures) Act 1986.

### Heart Excision and Perfusion

Adult male Wistar rats (275–325 g) were used for all perfusion experiments. Rats were co-administered sodium pentobarbital (200 mg.kg^−1^) and sodium heparin (200 IU.kg^−1^) by intraperitoneal injection. Hearts were excised and immediately arrested in ice-cold Krebs–Henseleit buffer (KHB). The aorta was cannulated and secured using 3–0 suture (Ethicon), the pulmonary artery incised to drain coronary effluent, and perfusion rate increased to (and maintained at) 14 ml.min^−1^. Contractile function was monitored with an intraventricular balloon set to an initial end-diastolic pressure of 4–10 mmHg. Perfusion pressure and cardiac contractile functional data were recorded using two pressure transducers connected to a PowerLab data acquisition system (AD instruments Ltd).

### Tracer Synthesis and Radiosynthesis

The syntheses of radiolabelling precursors and reference material and their characterisation and [^18^F]DHE radiosynthesis are described in the supplemental information.

### Determination of Radiotracer Lipophilicity

To measure the lipophilicity of both reduced and oxidised [^18^F]DHE, their octanol/PBS partition coefficients at pH7.4 (log D) were determined. 1 kBq of radiolabelled compound (10 μl) was added to 1 ml of a 1:1 mixture of PBS (pH7.4) and octanol, vortexed (1 min), and centrifuged (3000 × *g*), and an aliquot of each phase was measured by gamma counting. LogD was determined using the following equation:

$${LogD}_{7.4}={log}_{10}\left(\frac{\left[{}_{}^{18}F\right]{DHE}_{\left(octanol\right)}}{\left[{}_{}^{18}F\right]{DHE}_{\left({PBS}_{7.4}\right)}}\right)$$

### Determination of Radiotracer Chemoselectivity

Ten microlitres of [^18^F]DHE (100 kBq in PBS, pH7.4) was added to 990 μl of a range of ROS-generating systems: ethanol, PBS (pH 7.4), KO_2_ (1 mg.ml^−1^ in PBS, pH7.4), KO_2_ and ascorbic acid (1 mg.ml^−1^ and 1 mg.ml^−1^ in PBS, pH7.4), H_2_O_2_ (50 μM in PBS, pH7.4), Fe(III)Cl_2_ (1 mg.ml^−1^ in PBS, pH7.4), H_2_O_2_ and Fe(III)Cl_2_ (50 μM and 1 mg.ml^−1^ in PBS, pH7.4), vortexed (10 s), and incubated at room temperature for 5 min. One hundred microlitres of each aliquot was subjected to radioHPLC analysis, with percentage of radiotracer oxidation calculated using the ratio of the areas under peaks of the resulting chromatogram.

### The Triple ʏ-Detector System

Radiotracer pharmacokinetics were monitored using our custom built triple γ-detector system described previously [8–13]. This system comprises three orthogonally positioned lead-collimated Na/I γ-detectors arrayed around a Langendorff isolated heart perfusion rig. The detectors are sited: (i) 3 cm downstream of the injection port, 15 cm upstream of the heart cannula on the arterial line, (ii) directly opposite the heart, and (iii) over the venous outflow. Each detector was connected to a modified GinaSTAR™ ITLC system running Gina™ software for real-time data collection (Raytest Ltd, UK).

### Experimental Perfusion Protocol

After the stabilisation period, radiotracer in KHB was infused via syringe pump into the arterial inflow line at a constant rate of 50–150 kBq.min^−1^, its cardiac accumulation monitored using the triple-γ-detection system, and cardiac haemodynamics and perfusion pressure monitored using the Powerlab system. Perfusate supply was then switched to either 10 or 50 μM menadione in KHB, or vehicle (0.1% EtOH), while tracer infusion and perfusate flow rate were kept constant. The ratio between the accumulation rate before and after buffer switching in each case was calculated to normalise tracer uptake and allow comparison between experiments. Hearts were snap-frozen in liquid nitrogen and stored at − 80 °C prior to analysis for biomarkers of oxidative stress.

### Determination of Total Cardiac Glutathione

Tissue was homogenised into buffer containing Triton X-100 (0.1%, v/v), sulfosalicylic acid (5%, w/v), EDTA (5 mM), and potassium phosphate buffer (0.1 M, pH7.5) to yield a 10% homogenate (w/v). Total glutathione concentration was determined using an enzyme recycling method based on the quantification of 5,5′-dithio-bis-[2-nitrobenzoic acid] (DTNB) formation rate, measured at 412 nm [14].

### Measurement of Cardiac PKG1α Oxidation

Tissue was homogenised into buffer containing Tris–HCl (100 mM, pH7.4) maleimide (100 mM), and protease inhibitors (Roche, protease inhibitors complete EDTA free) to yield a 10% homogenate (w/v). Oxidation of PKG1α was determined by non-reducing gel electrophoresis with Western blotting using anti-PKG1α (Enzo Life Science (diluted 1:1000) and horseradish peroxidase linked anti-rabbit IgG (Cell Signalling Ltd.) diluted 1:1000 in blocking buffer [15]. PKG1α oxidation was determined by measuring the relative intensity of both monomeric (75 kDa) and dimeric (150 kDa) bands [16].

### Data Analysis

All analyses were blinded and randomised. Data are expressed as mean ± SD unless otherwise stated. If only two groups were compared, data were subject to a two-tailed unpaired Students t-test. One-way ANOVA was employed to compare single measures between multiple groups, with a Tukey post hoc test to account for multiple comparisons. Differences were considered statistically significant if *p* < 0.05.

## Results

**Fig. 2:**
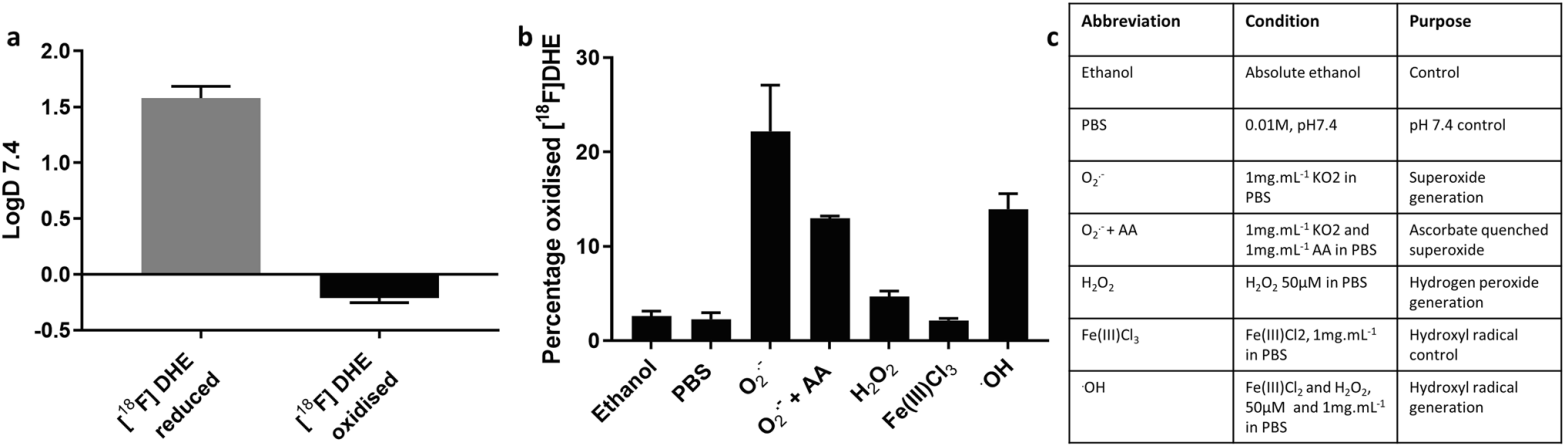
**A** LogD values of reduced (grey) and oxidised (black) [^18^F]DHE (mean ± SD, *n* = 6). **B** Selectivity of [^18^F]DHE for ROS (mean ± SD, *n* = 3). **C** Table to show conditions used for chemoselectivity measurements in panel **B**.

The logD of reduced [18F]DHE was 1.58 ± 0.11, which decreased to − 0.21 ± 0.04 when oxidised (Fig. 2). [^18^F]DHE (~ 1 MBq.ml^−1^) was sensitive to the following ROS generation systems: ethanol 2.25 ± 2.99%, PBS (0.01 M, pH7.4) 1.76 ± 2.76%, superoxide (1 mg.ml^−1^ KO_2_ in PBS) 18.74 ± 25.65%, superoxide and ascorbic acid (1 mg.ml^−1^ KO_2_ and 1 mg.ml^−1^ AA in PBS) 13.5 ± 12.8%, hydrogen peroxide (H_2_O_2_ 50 μM in PBS), iron chloride (Fe(III)Cl_2_, 1 mg.ml^−1^ in PBS) 2.3 ± 1.98%, and hydroxyl radical (Fe(III)Cl_2_ and H_2_O_2_, 50 μM and 1 mg.ml^−1^ in PBS) 15.1 ± 12.8% (Fig. 2).

**Fig. 3:**
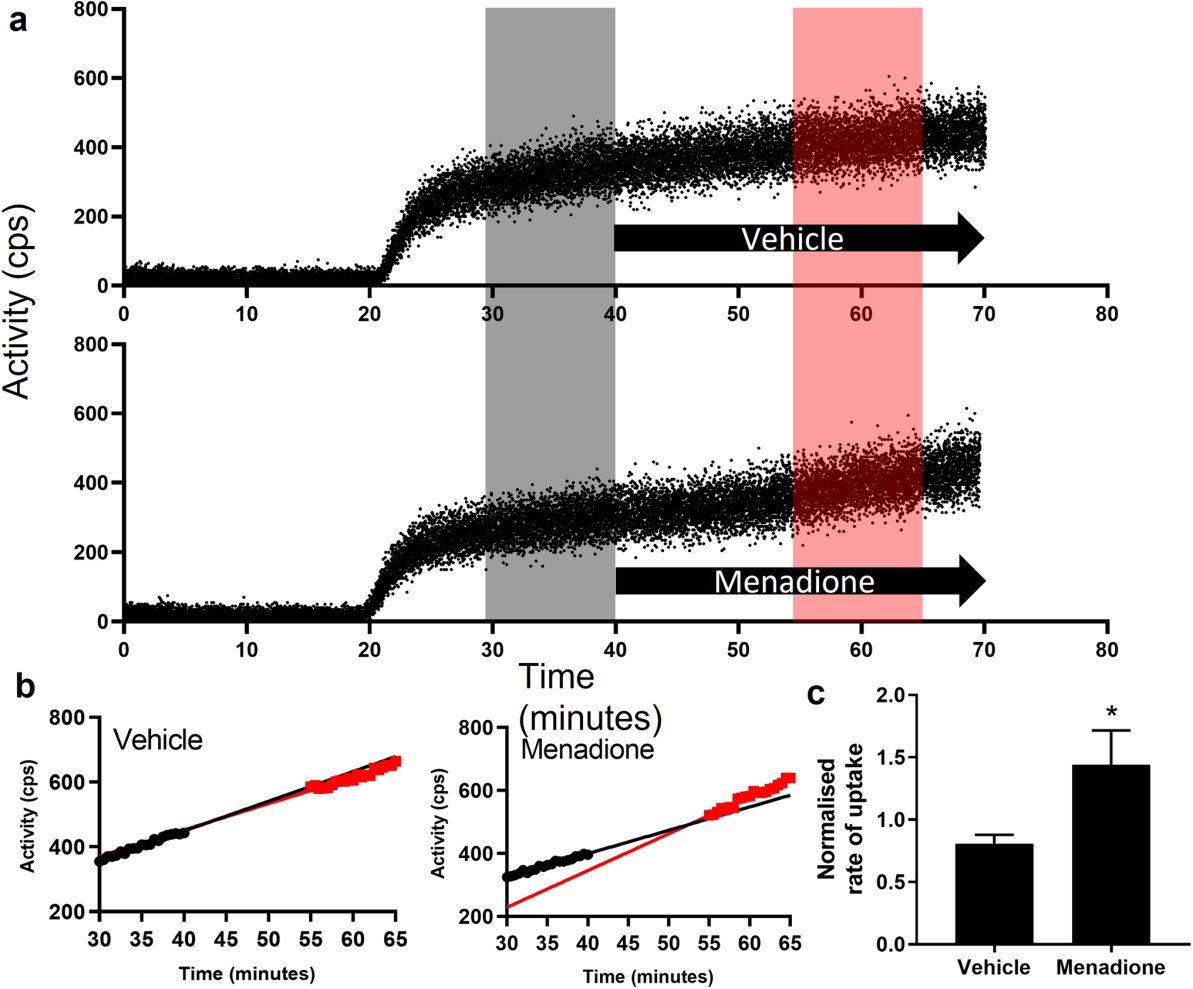
**A** Representative time-activity curves demonstrating [^18^F]DHE uptake in hearts perfused with vehicle or menadione (50 μM). Black arrow represents treatment, grey shows the baseline rate of uptake, and red shows the rate of uptake during treatment. **B** Representative trace of data used to normalise uptake of [^18^F]DHE over time during control conditions (30–40 min, black) versus vehicle (55–65 min, red). **C** Comparison of [^18^F]DHE uptake rate during control conditions and 50 μM menadione infusion (*n* = 4, ± SD, * = *p* < 0.05).

Representative outputs from the gamma detector interrogating isolated hearts are shown in Fig. 3A and fitted to uptake rates pre- and post-menadione infusion in Fig. 3B. The normalised rate of [^18^F]DHE uptake was significantly greater in menadione-treated hearts (1.44 ± 0.27) than was observed in vehicle control hearts (0.81 ± 0.07) (*p* < 0.05, *n* = 4/group, Fig. 3C).

**Fig. 4:**
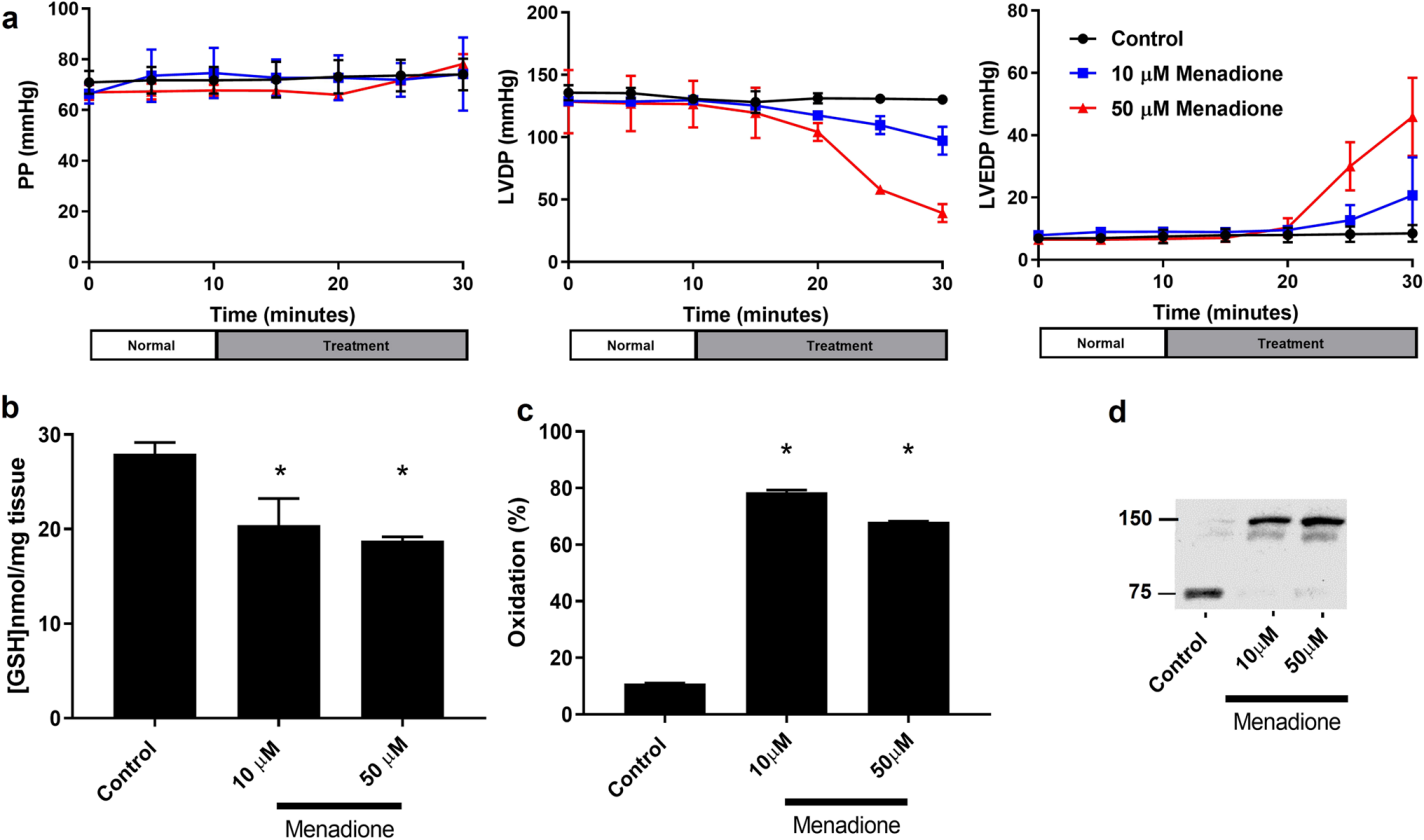
**A** Effect of menadione on cardiac function in isolated rat hearts. LVDP (top left), LVEDP (top right), PP (bottom left), HR (bottom right) (*n* = 4, ± SD). **B** Depletion of reduced glutathione by menadione administration. **C** Quantification of PKG1α oxidation by menadione administration. **D** Western blot used for quantification of PKG1α.

The effect of menadione infusion on cardiac haemodynamics is shown in Fig. 4A. Perfusion pressure was unaffected at any concentration used. LVDP progressively fell in a dose-dependent manner from 114.9 ± 17.1 mmHg (control) to 96.9 ± 11.4 mmHg (10 μM) and 39.2 ± 7.1 mmHg (50 μM). LVEDP progressively increased in a dose-dependent manner from 8.5 ± 2.7 mmHg (control) to 20.7 ± 8.6 mmHg (10 μM) and 45.9 ± 12.5 mmHg (50 μM). Heart rate remained stable in all groups at 340BPM. Menadione infusion caused significant depletion of tissue total glutathione content from 28.0 ± 1.2 (control) to 20.4 ± 2.8 (10 μM) and 18.8 ± 0.4 (50 μM), while PKG1α dimer formation increased significantly from 11.0 ± 0.1% (control) to 78.5 ± 0.9% (10 μM) and 68.1 ± 0.2% (50 μM) (*p* < 0.05, *n* = 6).

## Discussion

We have demonstrated the cardiac retention of the putative ROS-sensing radiotracer [^18^F]DHE in isolated perfused hearts confirmed to be generating ROS at the time of radiotracer administration. Further, we have shown that this increased retention occurs under conditions where perfusion was strictly maintained constant, to ensure as far as possible that this increased tracer retention was due to elevated ROS production rather than regional variations in radiotracer delivery and washout.

To induce ROS production, we utilised menadione, which redox cycles at the active site of flavoproteins to catalyse superoxide generation [17]. While prior studies evaluating radiolabelled DHE analogues have relied on historical association of their models with ROS generation [3, 4, 18], we have directly confirmed that tracer trapping coincides with demonstrable oxidative stress. We assayed our biomarkers in parallel time points coinciding with radiotracer infusion rather than assaying all hearts at the end of each protocol, which would otherwise overestimate the oxidative burden (being the summation of all the oxidative stress throughout the entire protocol, as opposed to just the period when the tracer was actually present). Under these conditions, total glutathione (GSH) levels (largely representing reduced GSH) were depleted, concomitant with increased PKG1α dimerisation, consistent with other such *ex vivo* models of oxidative stress [16, 19, 20].

Delivering [^18^F]DHE by constant infusion allowed us to probe the entire time-course of ROS generation by menadione infusion, which to some extent mitigates the potential problems associated with slow tracer trapping kinetics, as well as the challenge of determining exactly *when* maximal ROS generation occurs after the onset of menadione infusion (and in turn *when* to inject the tracer if a bolus approach were used). Even so, it must be appreciated that constant menadione infusion does not necessarily dictate a constant rate of ROS production; progressive oxidative damage may limit the capacity of the heart to continue to generate ROS as the experiment continues, while progressive depletion of antioxidants might mean that tissue oxidative stress could increase despite ROS generation falling.

Our lipophilicity measurements and chemoselectivity data support the proposed trapping mechanism, whereby the lipophilic and cell penetrant [^18^F]DHE becomes oxidised intracellularly by ROS to a cationic hydrophilic species unable to leave target cells, and we demonstrate that superoxide and hydroxyl radicals are capable of oxidising the radiolabelled probe. While our [^18^F]DHE analogue is not identical to those previously described in the literature (we have modified the 5-ethyl functionality of DHE rather than labelling via the 6-phenyl position), its selectivity to the various ROS species tested does not differ significantly from that of previously published probes. While this structural variation may cause subtle differences in the relative pharmacokinetic profiles of these analogues, the selectivities and trapping mechanism of all of these complexes is common, and we would suggest that our findings would be broadly representative of the general class of DHE-based ROS-sensing radiotracers.

While our highly controlled experiments do confirm ROS-dependent tissue trapping of [^18^F]DHE, it is perhaps not as profound as might be expected from prior *in vivo* studies with other structural analogues in this class. It is possible that regional changes in perfusion and vascular permeability may significantly alter the uptake and washout kinetics of tracers that trap by this mechanism *in vivo*. To extract meaningful insight from the regional retention of these tracers *in vivo*, we believe it is essential to correct for regional variation in radiotracer delivery and washout using independent parallel perfusion imaging [12]. As multiplexed imaging and pharmacokinetic approaches continue to evolve, it may soon be possible to serially or co-inject imaging agents to obtain such perfusion-corrected datasets to better understand and harness the potential of probes of this sort.

## Supplementary Information

Below is the link to the electronic supplementary material.

Supplementary file1 (DOCX 589 KB)

## References

[1] Carroll VN, Truillet C, Shen B (2016). [11C]Ascorbic and [11C]dehydroascorbic acid, an endogenous redox pair for sensing reactive oxygen species using positron emission tomography. Chem Commun.

[2] Carroll V, Michel BW, Blecha J (2014). A boronate-caged [18F]FLT probe for hydrogen peroxide detection using positron emission tomography. J Am Chem Soc.

[3] Boutagy NE, Wu J, Cai Z (2018). In vivo reactive oxygen species detection with a novel positron emission tomography tracer,18F-DHMT, allows for early detection of anthracycline-induced cardiotoxicity in rodents. JACC Basic Transl Sci.

[4] Chu W, Chepetan A, Zhou D (2014). Development of a PET radiotracer for non-invasive imaging of the reactive oxygen species, superoxide, in vivo. Org Biomol Chem.

[5] Robinson KM, Janes MS, Pehar M (2006). Selective fluorescent imaging of superoxide in vivo using ethidium-based probes. Proc Natl Acad Sci.

[6] Wilson AA, Sadovski O, Nobrega JN (2017). Evaluation of a novel radiotracer for positron emission tomography imaging of reactive oxygen species in the central nervous system. Nucl Med Biol.

[7] Huang L, Li Z, Zhang D (2018). Highly specific and sensitive radioiodinated agent for in vivo imaging of superoxide through superoxide-initiated retention. Anal Chem.

[8] Baark F, Shaughnessy F, Pell VR (2019). Tissue acidosis does not mediate the hypoxia selectivity of [64Cu][Cu(ATSM)] in the isolated perfused rat heart. Sci Rep.

[9] Handley MG, Medina RA, Mariotti E (2014). Cardiac hypoxia imaging: second-generation analogues of 64 Cu-ATSM. J Nucl Med.

[10] Medina RA, Mariotti E, Pavlovic D (2015). Identification of low-grade cardiac hypoxia by PET. J Nucl Med.

[11] Mariotti E, Veronese M, Dunn JT (2013). Assessing radiotracer kinetics in the Langendorff perfused heart. EJNMMI Res.

[12] Safee ZM, Baark F, Waters ECT (2019). Detection of anthracycline-induced cardiotoxicity using perfusion-corrected 99mTc sestamibi SPECT. Sci Rep.

[13] Shaughnessy F, Mariotti E, Shaw KP (2014). Modification of intracellular glutathione status does not change the cardiac trapping of 64Cu(ATSM). EJNMMI Res.

[14] Rahman I, Kode A, Biswas SK (2007). Assay for quantitative determination of glutathione and glutathione disulfide levels using enzymatic recycling method. Nat Protoc.

[15] Burgoyne JR, Eaton P (2013). Approaches for monitoring PKG1 a oxidative activation. Methods Mol Biol.

[16] Prysyazhna O, Burgoyne JR, Scotcher J (2016). Phosphodiesterase 5 inhibition limits doxorubicin-induced heart failure by attenuating protein kinase G Iα oxidation. J Biol Chem.

[17] Kumagai Y, Shinkai Y, Miura T, Cho AK (2012). The chemical biology of naphthoquinones and its environmental implications. Annu Rev Pharmacol Toxicol.

[18] Hou C, Hsieh CJ, Li S (2018). Development of a positron emission tomography radiotracer for imaging elevated levels of superoxide in neuroinflammation. ACS Chem Neurosci.

[19] Rainer PP, Kass DA (2016). Old dog, new tricks: Novel cardiac targets and stress regulation by protein kinase G. Cardiovasc Res.

[20] Haramaki N, Stewart DB, Aggarwal S (1998). Networking antioxidants in the isolated rat heart are selectively depleted by ischemia-reperfusion. Free Radic Biol Med.

