# Supplementary material for: Detecting Validated Intracellular ROS Generation with 18F-dihydroethidine-Based PET
Source: Mol Imaging Biol. 2021 Nov 24;24(3):377–83. doi: 10.1007/s11307-021-01683-0 (PMC9085669; doi:10.1007/s11307-021-01683-0)
Supplement: Supplementary file 1 — Supplementary file1 (DOCX 589 KB) [file 11307_2021_1683_MOESM1_ESM.docx]

**Electronic Supplementary Material**

**Detecting validated intracellular ROS generation with ^18^F-dihydroethidine-based PET**

**Journal: Molecular Imaging and Biology**

Edward CT. Waters^1^, Friedrich Baark^1^, Zilin Yu^1^, Filipa Mota^1,2^, Thomas R. Eykyn^1^, Ran Yan^1^, Richard Southworth^1^

^1^Division of Imaging Sciences and Biomedical Engineering, Kings College London, The Rayne Institute, St Thomas Hospital, London SE1 7EH, UK.

^2^Center for Infection and Inflammation Imaging Research, Center for Tuberculosis Research, and Department of Pediatrics, Johns Hopkins University School of Medicine, Baltimore, Maryland 21287, United States

### *Correspondence to:* Richard Southworth; *email:* *richard.southworth@kcl.ac.uk*

### General information

All reagents were purchased from Sigma-Aldrich unless otherwise stated. All gas mixtures were purchased from BOC Industrial Gases.

### Synthetic Chemistry

**3,8-bis(*tert*-butyloxycarbonylamino)-6-phenylphenanthridine (4)**

Di-*tert*-butyl dicarbonate (17.6 g, 81.2 mmol) was dissolved in EtOH (30 mL) to which guanidine hydrochloride (156 mg, 0.6 mmol) was added. 3,8-diamino-6-phenylphenanthridine (1.12 g, 3.9 mmol) was added to the stirred solution and the resulting mixture was stirred for 2 hours at 40 ^o^C. The progression of the reaction was monitored by TLC and once completed the product was obtained as a precipitate, separated by vacuum filtration, washed with ethanol and dried under vacuum to yield the title compound as a light-yellow precipitate **1** (1.64 g, 86.2% yield).

^1^H NMR (DMSO-d_6_, 400 MHz) δ 9.75 (s, 1H), 9.70 (s, 1H), 8.71 (d, 1H), 8.61 (d, 1H), 8.22 (s, 2H), 8.03 (d, 1H), 7.83 (d, 1H), 7.71 (d, 2H), 7.60 (m, 3H), 1.53 (s, 9H), 1.45 (s, 9H); ^13^C NMR (DMSO-d_6_, 100 MHz) δ 159.1, 151.6, 142.1, 138.4, 137.0, 128.5, 127.5, 127.0, 123.2, 121.9, 121.7, 121.4, 117.9, 117.2, 115.3, 113.9, 78.2, 27.0, 26.9; mp: 191.6-193.0 °C; LC-MS (m/z): C_29_H_31_N_3_O_4_ ([M+H]^+^) calc. 486.2388 found 486.2365.

**But-3-yn-1-yl trifluoromethanesulfonate (1)**

A solution of trifluoromethanesulfonic anhydride (10 g, 35 mmol) in dry DCM (12 mL) was stirred at 0 ^o^C under argon. To this a solution of 3-butyn-1-ol (2.1 g, 30 mmol) and dry pyridine (2.4 mL, 30 mmol) in dry DCM (12 mL) was added drop wise *via* syringe. A white precipitate of pyridinium salt formed upon addition. The reaction mixture was stirred at 0 ^o^C for 30 min, after which it was quenched with deionised water (10 mL). The organic layer was separated and washed with deionised water (10 mL x 2), brine (10 mL x 1), dried over MgSO_4_, filtered and concentrated in vacuo (200 mbar at 25^o^C) to give a light brown liquid (52% yield). The product was of sufficient purity and used immediately without further purification.

^1^H NMR (CDCl_3_, 400 MHz) δ 4.49 (t, 2H), 2.65 (td, 2H), 2.03 (t, 1H); ^13^C NMR (CDCl_3_, 100 MHz) δ 118.58 (q), 76.89, 73.60, 71.76, 19.87; ^19^F NMR (CDCl_3_, 400 MHz) δ -74.69; LC-MS (m/z): C_5_H_6_S_3_O_3_F+ ([M+H]^+^) calc. 202.9990 found 202.9381.

**5-(but-3-yn-1-yl)-3,8-bis(*tert*-butyloxycarbonylamino)-6-phenylphenanthridinium (5)**

Compound **7** (2.0 g, 10.0 mmol) was added to a suspension of NaHCO_3_ (840 mg, 10.0 mmol) and **1** (970 mg, 2.0 mmol) in nitrobenzene (30 mL). The reaction mixture was stirred for 24 hours and then nitrobenzene was removed by a short flash chromatography. The reaction mixture was loaded directly onto the column and eluted with DCM until all nitrobenzene was removed. The remaining material on the column was then eluted with (9:1 DCM/MeOH). The eluted fraction was then evaporated to dryness and loaded onto a new column. The desired compound was then eluted with (CH_2_Cl_2_, 50:1 CH_2_Cl_2_/MeOH, 20:1 CH_2_Cl_2_/MeOH 9:1 CH_2_Cl_2_/MeOH) to yield the title compound as an orange precipitate **2** (594 mg, 44% yield).

^1^H NMR (CDCl_3_, 400 MHz) δ 9.15 (s, 2H) 8.63 (d, 1H), 8.52 (d, 2H), 8.45 (d, 1H), 8.23 (d, 1H), 7.70 (m, 3H), 7.49 (m, 3H), 5.05 (t, 2H), 2.97 (t, 2H), 1.73 (s, 1H), 1.50 (s, 9H), 1.40 (s, 9H); ^13^C NMR (CDCl_3_, 100 MHz) δ 165.0, 154.8, 154.5, 144.5, 141.9, 135.4, 132.9, 132.5, 132.22, 130.9, 130.2, 126.8, 126.4, 124.5, 123.4, 123.2, 122.6, 120.2, 119.2, 107.1, 82.3, 81.9, 78.8, 74.2, 61.6, 53.7, 28.7, 28.6; mp: 166-174^o^C (decomposition) LC-MS (m/z): C_33_H_36_N_3_O_4_^+^ ([M+H]^+^) calc. 538.2701 found 538.2683.

**3,8-bis(*tert*-butoxycarbonyl)amino)-5-(2-(1-(2-fluoroethyl)-1*H*-1,2,3-triazol-4-yl)ethyl)-6-phenylphenanthridinium 6)**

To a solution of compound **2** (120 mg, 0.18 mmol) in DMF (1 mL), was added a solution of CuSO_4_·5H_2_O (38 mg, 0.15 mmol), bathophenanthrolinedisulfonic acid disodium salt hydrate (BPDS) (77 mg, 0.15 mmol) in phosphate buffer (0.1 M, pH = 7.4), compound **7** (5.0 equiv, 0.5 M solution in DMF), and sodium ascorbate (143 mg, 0.72 mmol) in H_2_O. The mixture was stirred at room temperature under N_2_ for 24 hours. The reaction mixture was then diluted with ethyl acetate and washed with brine (10 mL x 5) to remove the DMF. The organic layer was then dried with MgSO_4_, filtered and evaporated to dryness. The residue was then purified by SiO_2_ flash chromatography (CH_2_Cl_2_, 50:1 CH_2_Cl_2_/MeOH, 20:1 CH_2_Cl_2_/MeOH 9:1 CH_2_Cl_2_/MeOH) to yield the title compound as a red precipitate (91 mg, 65%).

^1^H NMR (CDCl_3_, 400 MHz) δ 8.77 (d, 2H) 8.69 (d, 1H) 8.04 (d, 1H) 7.85 (d, 1H) 7.78 (s, 1H) 7.68 (m, 4H) 7.37 (d, 2H) 4.91 (t, 2H) 4.62 (dt, 2H) 4.59 (t, 2H) 3.32 (t, 2H) 1.48 (s, 9H) 1.33 (s, 9H); ^13^C NMR (CDCl_3_, 100 MHz) δ 164.7, 154.8, 154.4, 144.5, 141.9, 135.5, 132.8, 132.5, 132.2, 130.7, 129.6, 126.9, 126.5, 124.5, 123.0, 122.8, 119.0, 83.7, 82.0, 55.2, 52.9, 28.7, 28.5, 25.7; mp 154-167^o^C (decomposition) LC-MS (m/z): C_35_H_40_FN_6_O_4_^+^ ([M+H]^+^) calc. 627.3090 found 627.2824.

**3,8-diamino-5-(2-(1-(2-fluoroethyl)-1*H*-1,2,3-triazol-4-yl)ethyl)-6-phenylphenanthridin-5-ium (7)**

Compound **3** (50 mg, 0.065 mmol) was dissolved in ethyl acetate (3 mL), to which conc. hydrochloric acid (1 mL) was added drop wise. The reaction was stirred at 40 ^o^C under N_2_ and monitored by TLC until completion. Ethyl acetate was removed under vacuum and then freeze dried to remove water. The resulting residue was washed with hexane and then dried under vacuum to yield the title compound has a dark red solid (40 mg, 95% yield).

^1^H NMR (CDCl_3_, 400 MHz) δ 8.97 (dd, 2H) 8.02 (m, 3H) 7.72 (m, 3H) 7.64 (d, 1H), 7.55 (d, 2H) 7.41 (s, 1H) 4.76 (d, 2H) 4.67 (s, 2H) 3.44 (s, 2H) 3.21 (m, 2H); ^13^C NMR (CDCl_3_, 100 MHz) δ 164.7, 137.7, 136.4, 133.1, 132.7, 132.0, 131.0, 129.9, 128.1, 126.3, 125.9, 123.6, 121.7, 105.8, 83.4, 81.7, 54.5, 53.2, 53.0, 24.9; LC-MS (m/z): C_25_H_24_FN_6_+ ([M+H]^+^) calc. 427.2041 found 427.2163.

**5-(2-(1-(2-fluoroethyl)-1*H*-1,2,3-triazol-4-yl)ethyl)-6-phenyl-5,6-dihydrophenanthridine-3,8-diamine (8)**

To a solution of compound **4** (10 mg, 0.015 mmol) in MeOH (0.5 mL) was added a solution of NaBH_4_ (20 mg) in MeOH (0.5 mL) drop wise. The resulting solution was then stirred under N_2_ for 10 minutes. The MeOH was then evaporated under a stream of argon. The resulting residue was then dissolved in degassed ethyl acetate and washed with H_2_O (1mL x3). The organic layer was dried with MgSO_4_, filtered and concentrated under vacuum to reduce the volume. The remaining solvent was then removed under a stream of argon resulting in pure title compound as a white powder (9 mg, 90% yield).

^1^H NMR (CDCl_3_, 400 MHz) δ 7.32 (m, 3H) 7.18 (m, 5H) 6.47 (d, 1H) 6.24 (d, 2H) 6.02 (d, 1H) 5.36 (s, 1H) 4.95 (m, 4H) 4.77 (dt, 2H) 4.68 (dt, 2H); ^13^C NMR (CDCl_3_, 100 MHz) δ 148.8, 137.3, 132.6, 132, 130.3, 129.3, 127.4, 126.9, 123.3, 121.3, 120.8, 118.9, 103.2, 81.7, 78.9, 74.6, 54.5, 49.1, 46.3, 25.7; LC-MS (m/z): C_25_H_26_FN_6_+ ([M+H]^+^) calc. 429.2197 found 429.2169.

**2-fluoroethyl 4-methylbenzenesulfonate (2)**

To a solution of 2-fluoroethanol (512 mg, 8.0 mmol) and pyridine (695 mg, 8.8 mmol) in dry DCM (3 mL/mmol 2-fluoroethanol) at 0^o^C was added a solution of tosyl chloride (1677 mg, 8.8 mmol) in dry DCM (7 mL) drop wise. The reaction mixture was warmed to RT and stirred for 4 h. The reaction was quenched by the addition of saturated NH_4_Cl (10 mL) and H_2­_O (10 mL). The mixture was extracted with CH_2_Cl_2_ (20 mL x3). The combined organic fractions were washed with brine, dried (MgSO_4_), filtered and concentrated in vacuo. The crude material was purified by flash chromatography on silica gel (hexanes: EtOAc, 50:50) affording 2-fluoroethyl-4-methylbenzensulfonate as a colourless oil (356 mg, 50% yield).

^1^H NMR (400 MHz, CDCl_3_) δ 7.80 (d, 2H), 7.35 (d, 2H), 4.57 (m, 2H), 4.26 (m, 2H), 2.45 (s, 3H). ^13^C NMR (100 MHz, CDCl_3_) δ 145.5, 133.3, 130.1, 128.6, 81.0, 68.7, 21.4; LC-MS (m/z) C_9_H_12_FO_3_S [M+H^+^]; calculated 219.0486, found 219.0492.

**1-azido-2-fluoroethane (3)**

Sodium azide (195 mg, 3 mmol) was added to a solution of compound **8** (178 mg, 2 mmol) in DMF (4 mL) with stirring overnight to form 1-azido-2-fluoroethane. The solution was filtered to yield the final compound as a 0.5M solution in DMF.

**3,8-diamino-5-(but-3-yn-1-yl)-6-phenylphenanthridin-5-ium (10)**

Compound **2** (100 mg, 0.19 mmol) was dissolved in ethyl acetate (3 mL), to which conc. hydrochloric acid (1 mL) was added drop wise. The reaction was stirred at 40^o^C under N_2_ and monitored by TLC until completion. The solvent was then removed under vacuum and then freeze dried to remove water. The resulting solid was washed with hexane and then dried under vacuum to yield the title compound as a dark red solid (86 mg, 95% yield).

^1^H NMR (CDCl_3_, 400 MHz) δ 8.78 (d, 1H) 8.73 (d, 1H) 7.76 (m, 5H) 7.62 (m, 2H) 7.42 (m, 2H) 4.83 (t, 2H) 2.37 (t, 2H) 2.05 (s, 1H); ^13^C NMR (CDCl_3_, 100 MHz) δ 165.0, 137.6, 136.5, 133.1, 132.72, 132.2, 131.6, 129.8, 128.0, 126.3, 126.3, 124, 125.3, 121.3, 120.6, 109.1, 83.9, 83.1, 81.2, 62.3, 54.1; LC-MS (m/z): C_23_H_20_N_3_+ ([M+H]^+^) calc. 338.1652 found 338.1584.

### Radiochemistry

**[^18^F]2-fluoroethyl azide (9)**

[^18^F]fluoride in H_2_O was trapped on an activated QMA cartridge (carbonate counter ion) and released with a mixture of kryptofix and potassium carbonate (30 mM:15 mM, 15% H_2_O in acetonitrile, 0.5 mL). The solvent was then removed by heating at 110^o^C under a stream of N_2_. The temperature was reduced to 90^o^C and anhydrous acetonitrile (0.4 mL) was added. The addition of anhydrous acetonitrile was repeated a further two times, ensuring the reaction vessel was evaporated to dryness each time. The vessel was then flushed with N_2_ to cool to 80^o^C before the addition of a solution of 2-azido-4-toluenesulfonate (1.8 mg in 0.4 mL anhydrous acetonitrile). The reaction was then heated at 80^o^C for 15 minutes, cooled to RT, quenched with H_2_O (1 mL) and purified by HPLC (zorbax SB-C18, 9.4 x 250 mm, 5 µm. Water(A)/acetonitrile(B) 0 min 20% B, 20 min 50% B, 30 min 80% B, 35 min 20% B. The flow rate was 2.5 mL/min, the compound eluted between 15.5-17 min (4 mL).

**3,8-diamino-5-(2-(4-(2-(fluoro-^18^F)ethyl)cyclopenta-1,3-dien-1-yl)ethyl)-6-phenylphenanthridin-5-ium (11)**

To compound **11** in HPLC eluent (4 mL, H_2_O/MeCN), was added compound **10** (0.5 mg, 0.89 µmol) in DMF (0.15 mL). To the resulting mixture was added a solution of CuSO_4_.5H_2_O (2 mg, 8.0 µmol) and BPDS (4.9 mg, 9.1 µmol) in phosphate buffered saline (0.1M, pH 7.4, 0.2 mL) and sodium ascorbate (7.9 mg, 39.9 µmol) in H_2_O (0.1 mL). The resulting solution was then capped and heated at 80^o^C for 30 minutes. It was filtered, the filter washed with H_2_O (0.5 mL) and purified by HPLC (zorbax SB-C18, 9.4 x 250 mm, 5 µm. Water 0.1% TFA (A)/methanol 0.1% TFA(B) 0 min 20% B, 20 min 50% B, 30 min 80% B, 35 min 20% B. The flow rate was 2.5 mL/min, the compound eluted between 26-27.5 min.

**[^18^F]5-(2-(1-(2-fluoroethyl)-1H-1,2,3-triazol-4-yl)ethyl)-6-phenyl-5,6-dihydrophenanthridine-3,8-diamine (12)**

A solution of **12** in HPLC eluent was diluted with H_2_O (10 mL). It was then loaded onto a C-18 cartridge and washed with H_2_O (10 mL). A solution of NaBH_4_ (20 mg) in water (1 mL) was then passed through the cartridge. The cartridge was immediately blown dry with air (10 cm^3^) and washed with sodium ascorbate solution (0.02 mg.mL^-1^, 10 mL). The cartridge was then eluted with a sodium ascorbate solution (0.02 mg.mL^-1^) of water and ethanol (20:80, 0.4 mL). The first 0.1 mL was discarded, and then the next 0.3 mL containing a constant concentration of compound **12**. This solution was then diluted with sodium ascorbate (0.02 mg.mL^-1^) in saline (0.9% w/v) to give a final volume of 2.4 mL, containing [^18^F]DHE, sodium ascorbate (0.02 mg.mL^-1^), ethanol (10%) and NaCl (0.9% w/v).

### HPLC chromatograms

[^18^F]2-fluoroethylazide

Radioactivity and UV absorbance (254 nm) chromatograms from HPLC purification of the [^18^F]2-fluoroethylazide synthon.

[^18^F]11

Radioactivity and UV absorbance (254 nm) chromatograms from HPLC purification of [^18^F]11.

|   [^18^F]DHE  [^18^F]impurities    Ascorbate  Radioactivity and UV absorbance chromatograms (254 nm) from QC run of [^18^F]DHE, region 1 = 95.5%, region 2 = 4.5%.UV signal at 2-5 min due to ascorbate. A purity of greater than 95% was deemed acceptable for use of the tracer in further experiments. The total synthesis time was 2 hours and 15 minutes from end of bombardment. The non-corrected radiochemical yield of the synthesis was 14.1 ± 2.3% and the decay corrected yield was 33.1 ± 5.4% (n=14). |
| --- |

Overlaid radioactivity and UV absorbance (254 nm) chromatogram from injection of [^18^F]DHE (blue, cps), with [^19^F]DHE spike (red, mAU).

### Molar activity measurement

Calibration curve of compound **8** for calculation of molar activity of [^18^F]DHE (n=3). The limit of detection of compound **8** was 2.33 ng (all samples were injected in a volume of 20 μL in a 10% solution of ethanol in water). The calibration curve was used to calculate the molar activity of a 25MBq aliquot of [^18^F]DHE. The molar activity was calculated to be 72 GBq.µmol^-1^.

### Chemoselectivity study

|   B  A    F  E  D  C    H  G    I  Representative HPLC chromatograms of [^18^F]DHE after incubation with different ROS or control conditions: (A): Ethanol, (B): PBS, (C): superoxide, (D): superoxide and ascorbic acid, (E): hydrogen peroxide, (F):iron chloride, (G): hydroxl radical, (H): PBS and (I): superoxide. A-G: 5 minute incubations, H&I: 1 hour incubations. Green region: reduced [^18^F]DHE, red regions: oxidation products of [^18^F]DHE. |
| --- |
